# Experiences of healthcare providers caring for pregnant individuals with substance use disorder

**DOI:** 10.1016/j.drugalcdep.2025.112942

**Published:** 2025-10-30

**Authors:** Assumpta Nantume, Kylie Leong-Bob, Olivia R. Hanson, Jami Baayd, Connie Wilson, Alexandra Gero, Karen W. Tao, Rebecca G. Simmons, Marcela C. Smid, Erin P. Johnson, Torri D. Metz, Melissa H. Watt, Susanna R. Cohen

**Affiliations:** a ELEVATE Center, Department of Obstetrics and Gynecology, University of Utah Health, United States; b Spencer Fox Eccles School of Medicine at the University of Utah, United States; c ASCENT Center, Department of Obstetrics and Gynecology, University of Utah Health, United States; d Department of Nursing, Utah State University, United States; e Department of Educational Psychology, University of Utah, United States; f Department of Population Health Sciences, University of Utah, United States

**Keywords:** Substance use disorder, Respectful maternity care, Perinatal substance use, Addiction, Provider bias, Provider stigma, Provider training

## Abstract

**Introduction:**

Substance use disorder (SUD) during pregnancy is associated with an increased risk of adverse maternal and neonatal outcomes, yet many patients face significant barriers to accessing treatment, including experiencing bias and stigma from healthcare providers. To inform improvements in care delivery, this study explored the experiences of healthcare providers who care for pregnant individuals with SUD.

**Methods:**

Researchers conducted seven focus group discussions (FGD) and fifteen in-depth interviews (IDI) using semi-structured guides, with participants drawn from both rural and urban hospital settings across Utah. All discussions were audio recorded, transcribed verbatim, and examined using the Template Analysis approach.

**Results:**

Among FGD participants (n = 37), the sample was predominantly white (94.6 %), female (86.5 %), rural (89.2 %), and comprised of nurses (78.4 %). The IDI sample (n = 15) was more gender diverse (60 % female), had greater representation of physicians (53.3 %), and a higher proportion of urban participants (60.0 %).

Template analysis revealed four major themes. First, providers held a range of perceptions toward pregnant individuals with SUD, reflecting both stigma and empathy. Second, many emphasized the importance of building trust through nonjudgmental communication and emotional support. Third, providers reported high levels of burnout, particularly due to limited resources and systemic barriers. Finally, participants highlighted knowledge gaps related to SUD clinical care and confusion around regulatory requirements like mandatory reporting.

**Conclusions:**

Despite the challenges described, many providers expressed strong dedication to delivering compassionate, person-centered care. The findings underscore the need for targeted provider education, institutional policies that reduce care barriers, and increased community and institutional resources to better support patients with SUD during pregnancy.

## 1. Introduction

Substance use disorder (SUD), defined as a medical condition characterized by the compulsive use of substances despite harmful consequences, during pregnancy represents a pressing public health challenge in the United States, contributing to rising maternal morbidity and mortality (Smid et al., 2024; Davis et al., 2021) and adverse infant health outcomes (Oni et al., 2022a). According to data from the National Survey on Drug Use and Health (NSDUH), in 2023, between 8.4 % and 11.0 % of pregnant women aged 15–44 reported using illicit drugs, tobacco products, or alcohol in the past month (Substance Abuse and Mental Health Services Administration, 2024). SUD during pregnancy is associated with an increased risk of poor perinatal outcomes, including preterm birth, low birth weight, and neonatal abstinence syndrome (NAS) (Oni et al., 2022a; Chokshi et al., 2024). Despite the availability of treatment options, many pregnant individuals with SUD face barriers to accessing appropriate care, including social stigma and fear of legal repercussions (Burgos and Gelband, 2024).

A 2007–2016 national survey of hospital-based deliveries found that rural communities experience a higher prevalence of SUD during pregnancy compared to urban communities (24 % vs. 14 %) (Jarlenski and Krans, 2021). These disparities are compounded by geographic isolation, limited healthcare infrastructure and chronic workforce shortages that make it more difficult to treat and manage SUD in rural areas (Brooks et al., 2017; Davis and O’Neill, 2022; Kiang et al., 2021; Lister et al., 2020; Heitkamp and Fox, 2022). Populations living in rural areas often face heightened stigma surrounding substance use, driven by close-knit community dynamics, cultural attitudes, and limited privacy in seeking care (Thomas et al., 2020; Franz et al., 2021). These factors create an environment in which individuals with SUD may experience intensified judgment and reduced opportunities for anonymous or confidential treatment, further discouraging individuals from accessing necessary support (Heitkamp and Fox, 2022).

The challenges of addressing SUD are particularly pronounced among pregnant individuals, who face a complex intersection of stigma, medical vulnerabilities, and societal expectations for pregnancy and parenting of newborns (Weber et al., 2021a). Pregnancy introduces heightened scrutiny of behavior, particularly regarding substance use, with societal norms often framing substance use during pregnancy as a moral failing rather than a medical condition requiring compassionate care (Terplan et al., 2015a). Pregnant individuals with SUD are frequently subject to increased judgment, both in the broader social context and within healthcare settings, where they may encounter provider bias and discrimination (Merritt et al., 2022a). Fear of punitive actions, such as involvement of child protective services (CPS) or loss of custody, often drives avoidance of prenatal care, compounding risks to both maternal and fetal health (Oni et al., 2022b).

Systemic barriers, including lack of reliable transportation, unstable housing, and limited health insurance, further impede access to regular prenatal and addiction care (Bellerose et al., 2022; Kramlich et al., 2018). These challenges are magnified in rural areas, where long travel distances to healthcare facilities, shortages of obstetric and addiction specialists, and underfunded healthcare systems constrain access to timely and comprehensive care (Chan et al., 2006; Nesbitt et al., 1989; Lander et al., 2013). For pregnant individuals with SUD living in rural communities, these intersecting barriers create a uniquely precarious situation, placing them at increased risk of poor maternal and neonatal outcomes (Allen et al., 2024).

Despite these obstacles, rural healthcare providers often demonstrate remarkable adaptability, resilience, and dedication to delivering high-quality, compassionate care under challenging circumstances (Lasky et al., 2024; Chertok et al., 2023). By cultivating strong patient-provider relationships and leveraging community resources, rural providers play a critical role in addressing the health inequities faced by pregnant individuals with SUD (Kramlich et al., 2018; Bright et al., 2022). However, these efforts are often constrained by systemic barriers, such as limited funding, high patient-to-provider ratios, absence of specialized clinical providers, and insufficient support for professional development and training (Lander et al., 2013; Maganty et al., 2023). Additionally, the lack of comprehensive community-based services for SUD treatment and social support further limits referral options, making it more difficult to ensure continuity of care and long-term recovery support (Choi et al., 2022 Jun).

This study aims to explore the experiences and perspectives of healthcare providers caring for patients with SUD during the intrapartum period. By examining the challenges and strategies encountered by these providers, this research aims to illuminate the broader systemic and structural factors that shape the delivery of care in rural settings to facilitate improved maternal and neonatal outcomes in this vulnerable patient population.

## 2. Methods

### 2.1. Study design

This qualitative study which included focus group discussions (FGDs) and in-depth individual interviews (IDIs) was approved by the University of Utah Institutional Review Board (IRB #00167800). Data were collected as part of the larger Project INSPIRE (Interprofessional Simulation Program for Clinical Resilience and Empathy), which aims to develop and evaluate a multi-component training intervention to improve intrapartum care for individuals with SUD (Cohen et al., 2025).

### 2.2. Setting and participants

The study was conducted in ten clinical settings across Utah, including three urban tertiary care medical centers and seven rural community hospitals, with oversampling among facilities serving rural communities. The state of Utah is unique in that over 98 % of its landmass is classified as either rural or frontier (Utah Division of Data, 2025). Many of the rural communities in which this study was conducted experience geographic isolation, underscoring the challenges of accessing healthcare services and the importance of understanding provider experiences in these settings.

Eligible participants included healthcare team members, both medical (e.g., nurses, physicians, midwives, pharmacists) and non-medical (e.g., social workers, case managers, peer recovery specialists), who interacted with individuals during the intrapartum period. Healthcare team participants were recruited from various departments, such as emergency departments (EDs), labor and delivery (L&D), postpartum units, neonatal intensive care units (NICUs), and pediatrics. Using purposive sampling, we recruited eligible participants FGDs and IDIs by direct email invitations. All providers approached participated and received a $50 gift card for their time and insights.

While we established a relationship with the study sites prior to study commencement, interviewers had no direct relationship with the study participants. Participants were informed that the research team was interested in understanding provider experiences caring for patients with SUD. Participants were informed about the research team’s credentials, motivations, assumptions and funding sources.

### 2.3. Data collection

Data were gathered using a combination of FGDs and IDIs with unique participants, to capture the depth and complexity of healthcare providers’ experiences, with FGDs offering insight into shared team dynamics and IDIs allowing for individual perspectives on sensitive topics. Three experienced researchers (OH, JB, AN) facilitated the FGDs and IDIs using semi-structured interview and discussion guides that included open-ended questions and case scenarios. Guides were pilot tested and refined. Only participants and interviewers were present during sessions.

FGDs were conducted with healthcare teams in seven of the ten study sites between February and April 2024. Each FGD lasted approximately 90 min, exploring topics such as attitudes toward patients with SUD, prior training on SUD, availability of resources, principles of respectful maternity care (Miteniece et al., 2023), and team burnout, resilience, and support strategies. While the FGDs focused primarily on the intrapartum period, discussions often encompassed the broader perinatal period, from pregnancy through the late postpartum period. FGDs were instrumental in understanding group dynamics, shared experiences, and collective attitudes. One focus group was conducted virtually via Zoom to accommodate participant schedules, while the remaining six were held in person at the clinical sites.

Following completion of the FGDs, IDIs were conducted to delve into nuanced and sensitive topics, targeting a wider range of provider types, including anesthesiologists, pediatricians, psychiatrists and social workers, identified through our professional networks. We conducted all IDIs virtually via Zoom between May and September 2024. Interviews explored issues such as implicit biases affecting clinical decision-making, leadership’s role in shaping team culture, and barriers to implementing equitable, person-centered care. There were no repeat interviews, and none of the IDI participants had previously taken part in FGDs, ensuring fresh perspectives rather than extended discussions from prior participants. However, some IDI participants worked in the same facilities as FGD participants, allowing us to compare individual and group perspectives on shared institutional challenges. Interviews provided participants with a confidential space to share candid insights that may not have surfaced in group settings.

Thematic saturation was achieved by the time data collection concluded.

### 2.4. Data analysis

FGDs and IDIs were audio recorded and transcribed verbatim. Thematic analysis was performed using the Template Analysis method (Brooks et al., 2015), which provided a structured yet flexible approach to examining the data. Analyses were conducted using MAXQDA (ver 24.4.1). Two research team members (JB, OH) read all focus group transcripts and then collaboratively developed an initial coding template based on the study’s objectives and the emerging themes. This single template was used for both focus group and interview data. Next, four members of the research team (JB, OH, AN, KLB) collaboratively coded a subset of focus group and interview transcripts, further revising the coding template and establishing shared definitions of the codes. The remaining transcripts were collaboratively coded by two members of the research team (AN, KLB). Together, they iteratively refined the template to include new themes and subthemes as they emerged during the coding process. Coding discrepancies were resolved through regular team meetings in which coders discussed interpretations and reconciled differences by consensus. While we did not formally calculate inter-coder reliability coefficients, we prioritized reflexive dialogue and team consensus to enhance analytic rigor and ensure the validity of the findings. See Supplementary Materials for the final template. The systematic, yet flexible, approach of the Template Analysis method ensured rigor and reliability in the coding process and facilitated a comprehensive understanding of the multi-level factors influencing care for birthing individuals with SUD. Before our final reporting, findings were shared with participants for further validation and feedback.

### 2.5. Reflexivity statement

The study team was composed of interdisciplinary researchers with backgrounds in obstetrics, public health, nursing, and equitable health care for marginalized communities. These broad experiences informed the development of the interview guide and interpretive lens. To mitigate potential bias, all interviews and focus groups were conducted by trained qualitative researchers who had no direct supervisory or clinical relationship with participants. We use the term ‘substance use disorder (SUD)’ throughout this manuscript in alignment with person-first and non-stigmatizing language guidelines. Stigmatizing terms such as ‘substance abuse’ are retained only when directly quoted from participants or published sources.

## 3. Results

### 3.1. Description of the sample

Overall, 52 healthcare providers participated: 37 in focus groups and 15 in interviews. Of the 37 focus group participants, the majority were female (86.5 %), identified as white (94.6 %), and worked as nurses (78.4 %). Most focus group participants practiced in rural settings (89.2 %). The IDI sample (n = 15) was more gender diverse (60 % female), had greater representation of physicians (53.3 %), and a higher proportion worked in urban settings (60.0 %) (Table 1).

Four themes were identified in healthcare providers’ experiences caring for pregnant patients with SUD: 1) perceptions of pregnant individuals with SUD, 2) building trust through supportive communication and empathy, 3) provider burnout amid resource scarcity and systemic barriers, 4) knowledge gaps in clinical management and SUD-related regulations.

#### 3.1.1. Theme 1: perceptions of pregnant individuals with SUD

Healthcare providers spoke openly about how stereotypes and judgement towards pregnant individuals with SUD can affect the quality of care they receive. Providers grappled with their own judgements and also shared how they saw biases among their colleagues and within the healthcare systems. Providers described instances where these biases manifested in clinical decision-making, particularly in the context of pain management. Providers’ perception that patients might be medication-seeking, or providers’ fear of contributing to a patient’s SUD, often led to hesitation in prescribing pain medication, even when clinically indicated.

“I still see a lot of moralization of individuals with substance use disorder in the hospital. Where someone comes in and they have a history of opioid use, providers approach their pain management in very erroneous ways: If I give this patient pain medication, I’m contributing to their addiction.’ I always teach my residents, if a patient is having acute pain, maybe their pain is real, maybe it’s not, give everybody the benefit of the doubt.” FGD, urban hospital #1

“There are so many biases that clinicians bring to the table when they’re treating somebody with a substance use disorder. There’s this term we throw around, ‘med-seeking’, that I just think needs to go because when I go to my doctor, I’m usually med-seeking… There is a population without an opioid use disorder who need pain control, and a group of people with opioid use disorder that need pain control. They both get undertreated in different ways. The people with an opioid use disorder often just have opioids avoided altogether.” IDI-07, psychiatrist, urban hospital

Other providers acknowledged that their instinct to prioritize the baby’s well-being could unintentionally reinforce biases toward pregnant individuals with SUD. Many described a deeply-rooted moralization of substance use during pregnancy, where judgments about a patient’s choices often shaped clinical interactions and decision-making. One provider reflected on this moral tension, questioning how a pregnant individual could continue substance use despite knowing the potential harm, while others emphasized that their role was limited to ensuring a safe delivery rather than addressing broader social and behavioral complexities.

“I think part of the stigma is that the patient is pregnant and using. A lot of providers will feel like: Why can’t you stop? Why are you doing this to your baby? You’re not taking care of yourself; you’re not taking care of a human that is totally dependent on you. What kind of a person are you? You don’t deserve good care, because look at what you’re doing?” FGD, rural hospital #3

“Our job is to get the baby safely delivered. We can’t fix her substance abuse problem, we can’t fix her social problems, we can’t fix her history, we can’t fix her emotional problems. Our job is to safely deliver [her baby].” FGD, rural hospital 1

“I would like to understand how can they do this to their baby? I know it’s an addiction, but that’s the hard part for me. You have a little baby in there and you’re hurting it. And I know that’s not even what they think about. They need that addiction.” FGD, rural hospital #4

#### 3.1.2. Theme 2: building trust through supportive communication and clinical empathy

While not shying away from the discussion of bias towards pregnant individuals with SUD, many providers also expressed deep commitment to building patient trust through supportive communication. Providers emphasized the use of destigmatizing language and avoiding harmful terminology such as “clean” or “addict” which imply a moral component in substance use disorder. This person-centered approach was also reflected in how providers discussed patients with other providers, including avoiding biased language when giving reports to the oncoming shift.

“I used to say substance abuse, and I’ve learned that we don’t say ‘abuse’ because abuse has a negative connation to it so we say ‘substance use disorder.’ And always using person-first language is important, like ‘person with substance use disorder,’ just to be able to better communicate. We don’t say ‘addicted babies’ because addiction is a maladaptive behavior. Babies are born dependent.” FGD, urban hospital #1

Additionally, many providers actively worked to counter biases through conscious reflection, as one explained:

“My first inclination is always, ‘Oh, my gosh, you’re pregnant and you’re smoking pot?! You’re pregnant and you’re doing that?’ And I have to stop and remind myself what the patient’s life is like. I have to make a conscious choice to be compassionate, because my initial response is always anger.” FGD, rural hospital #2

Providers acknowledged that building trust with individuals with SUD was challenging due to entrenched mutual mistrust stemming from past negative healthcare experiences and that maintaining empathy was central to overcoming this barrier. Providers sought to convey empathy both in their direct interactions with patients and in discussions about their care, often reflecting on their patients’ broader life experiences beyond substance use disorder, including past trauma, financial struggles, shame, guilt and stigmatization. In many cases, providers cited personal or professional experiences that contributed to their ability to empathize with patients. Having people in their own lives who had SUD motivated providers to support their patients and advocate for a greater understanding of their patients’ backgrounds. In their interactions with patients, providers mentioned the benefits of just lending an ear to listen to patients and treat them with compassion and patience.

“It goes a long way to just have a positive interaction with someone. Especially when you’re in the medical system, and maybe someone’s only had years and years of negative interactions. And that’s not nothing. That physically and emotionally is good for people. And so, I don’t know, maybe that’s something that providers can learn too. You don’t need to fix the patient. But you can be a nice person who listens for a few minutes. And that might make a world of difference to that person.” IDI-12, nurse, rural hospital

Another key barrier to building patient trust was patients’ fear of legal consequences, particularly involvement of CPS. Providers mentioned situations where the patient avoided receiving care altogether due to fears that accessing the healthcare system would lead to their baby being removed from their custody. When patients with SUD did receive care, they were often worried about CPS, making them reluctant to open-up about their history with SUD and communicate transparently with their providers. Providers also expressed dismay that individuals working for CPS or law enforcement were not trained in trauma-informed care, and that they often resorted to punitive measures that further contributed to patients’ justified skepticism of the healthcare system.

“It’s so hard to get your kids back once you lose them. Once DCFS (Department of Child and Family Services) is involved… Sometimes they’re great, sometimes they’re not… But I don’t think they’re very trauma-informed or educated on substance abuse at all to be honest with you… they need a lot more education when it comes to dealing with situations like this.” IDI, peer support specialist, urban hospital

“I think we underestimate the stress and pain that the DCFS process causes to the mom and any other family members who might be involved. This is not just a benign somebody showing up trying to help you… I would love if we could improve it. Make the process really need-focused, rather than punitive. We should ask, ‘Okay, what do you need to parent this child effectively?’ Rather than, ‘What are you doing wrong?’” IDI-07, psychiatrist, urban hospital

“I have to tow the line and follow the law as closely as it’s written, but there are times when I wonder, ‘Are we really doing the right thing getting DCFS involved?’” IDI-07, psychiatrist, urban hospital

To foster trust and alleviate patients’ concerns, providers employed specific communication strategies that emphasized transparency and reassurance. This included keeping the patient informed at each step in the care process and proactively addressing any anxiety about potential legal consequences and CPS involvement. They also set realistic expectations for the patient regarding labor and pain management and ensured that the patient felt cared for. For instance, one provider elaborated on the complexities of managing pain for patients with SUD and his efforts to communicate that effectively:

“If I do a C-section on a patient [with SUD], I give all of them a speech about how the long-term use of opioids causes down-regulation of the nociceptor… which basically makes all painful stimuli more painful to them and it also means it’s really hard to manage that pain… So that does pose a challenge and I spend a lot of mental energy kind of managing the expectation for the patient, not that we don’t care about her pain, but it’s going to be really difficult to manage her pain in a safe way once her anesthesia has worn off.” IDI-03, physician, urban hospital

#### 3.1.3. Theme 3: provider burnout amid resource scarcity and systemic barriers

Providers acknowledged that maintaining empathy while facing high patient volumes and limited resources often led to emotional fatigue and burnout. Providers pointed to the heightened physical and psychological needs of patients with SUD, which greatly increased providers’ stress when combined with a lack of resources. They expressed frustration at the scarcity of specialized services, such as clinical specialists, residential treatment programs, mental health services, and substance use counseling, often forcing them to manage complex patient needs beyond their expertise.

“We don’t have a NICU team. We have family medicine providers, CRNAs [certified registered nurse anesthetist], and then just our regular nurses. So, we don’t have anyone that specializes in any of that [neonatal abstinence syndrome and addiction medicine], nor do we have the equipment.” FGD, rural hospital #5

“You have one OB (obstetrics) nurse, and your ER (emergency room) nurse is also your baby nurse. Because we don’t have the patient load, we don’t have the county funds, we don’t get government money to subsidize us… And that places a burden on the nurses. Our nurses probably suffer more than most, because they don’t have all the tools and resources. And the patients suffer too.” FGD, rural hospital #5

The challenge of resource scarcity also extended to staffing shortages, which strained providers’ capacity to offer patient-centered care.

“It does really affect your ability to be an empathetic, caring nurse because you’re so focused on tasks. You have so much to get done in a rural facility that I feel like in a big place, you probably could be more caring.” FGD, rural hospital #1

Providers also described facing additional barriers due to geographic isolation in rural settings. Many struggled with the reality that they were sending patients home without meaningful links to community services that could support long-term recovery. Without local rehabilitation centers, outreach programs, or specialized SUD treatment options, providers felt their patients were left with limited pathways to sustained recovery.

“I don’t think there’s enough support out here or drug treatment programs for pregnant women… And I think that’s another obstacle for patients that do deliver here. No rehab facilities or outreach programs that can come here.” FGD, rural hospital #4

“If any of these moms need rehab, we don’t have anything close. Now they have a new baby and are trying to find a rehab center that’s 5 h away, the likelihood of them going is very slim.” FGD, rural hospital #3

“There aren’t any programs here, there aren’t any places for them to go for help. So, if this mom does have a history of SUD… what resources do we have to give her? We have none, other than the social worker, and I have no idea what resources they have. But as far as I’m aware, I don’t know of anything in our community [that serves as] a support system for them, or anything other than trying to get them to go to counseling. And, again, we live in a very unique area… Our patients don’t have a lot of money and they don’t have a lot of resources, and abuse is rampant… There’s so much that we deal with down here.” FGD, rural hospital #5

These systemic barriers often left providers feeling disempowered and frustrated, and in some cases they even considered leaving their current positions to pursue less emotionally taxing opportunities. To mitigate burnout, many providers developed resiliency strategies to maintain well-being in the face of stress. Providers described self-care activities such as physical exercise, spirituality and meditation. They also described how they relied on their colleagues for support in challenging work environments, with some providers likening their colleagues to family. Providers expressed a desire for structural approaches to increase resiliency and mentioned organized debrief sessions as a possible outlet for employees to openly express their struggles and receive advice from peers. To reduce the burnout stemming from the lack of SUD treatment resources, providers reminded themselves of the positive influence that they had had on their patients. They expressed feeling reassured that they were able to be part of even a small change for the better in their patients’ lives.

#### 3.1.4. Theme 4: knowledge gaps in clinical management and sud-related regulations

Providers consistently reported uncertainty regarding best practices for treating patients with SUD, most notably in the areas of pain management during birth and postpartum, and the treatment of NAS in newborns. Many expressed concerns about balancing adequate pain relief with concerns over substance misuse and potential “medication-seeking” behavior. Some providers acknowledged that limited training and knowledge gaps on SUD-specific care left them feeling underprepared.

“We really don’t know what the best treatment plan is. Some clinicians can be very conservative in their willingness to give pain medicine in the acute period of labor and post operative pain. The nature of research is it is predominantly done in healthy, uncomplicated people. And any study that’s looking at pain is going to exclude patients with an opioid use disorder. They’re going to exclude patients with mental health problems. And I think that is a knowledge gap that would be really, really beneficial to know.” IDI-13, anesthesiologist, urban hospital

The management of substance-exposed neonates, including those affected by NAS, also emerged as a significant knowledge gap, with providers emphasizing their limited experience.

“Our nurses are not experienced in treating babies with NAS. I think everyone’s a little out of their comfort zone with babies with NAS because we don’t get a lot of babies who have NAS. And because if we needed to transfer a baby to a different hospital because they were not doing well, that process can take 3–4 h sometimes. So we’re always a little bit worried about [admitting] any pediatric patient who may rapidly decompensate… if we think that there’s a decent chance that they’re going to need higher level care.” IDI-11, physician, rural hospital

In addition to clinical uncertainties, providers also reported confusion regarding legal and policy considerations surrounding perinatal substance use. Specifically, several participants noted ambiguity regarding mandatory reporting laws and institutional guidelines for testing and treatment of patients with SUD during the intrapartum period, leading to inconsistent practices and potential disparities in care.

“What I feel would be valuable is if we had policies, procedures, or guidelines on best practices for a mom that might be using meth, a mom that might be using cocaine, a mom that might be using Suboxone… If we don’t know the patient has a substance use disorder, but we’re seeing these [potential withdrawal signs] in an infant. What are those signs and symptoms, what do they look like? And what should those next steps be? As much as we feel like we might know the legal reporting requirements, I think that that would be a good refresher to go over what are the actual legal requirements of us as clinicians.” FGD, rural hospital #5

## 4. Discussion

This study explored the experiences and perspectives of healthcare providers caring for pregnant individuals with SUD. Four key themes emerged: perceptions of pregnant individuals with SUD, the importance of trust-building through empathy and communication, provider burnout amid systemic barriers, and knowledge gaps in clinical management and SUD-related regulations, particularly those concerning mandatory reporting requirements. These findings underscore the complex interplay of personal, institutional, and structural factors influencing care delivery for this vulnerable population, and point to opportunities to support healthcare providers, especially those in rural areas, to provide quality, respectful care that improves patient outcomes.

Consistent with previous research (Merritt et al., 2022b; Terplan et al., 2015b; Crawford et al., 2022; Goodman, 2024), this study highlights the pervasive impact of stigma and bias in healthcare settings. Providers described instances where personal bias influenced clinical decision-making, particularly in pain management. This aligns with existing literature suggesting that individuals with SUD often receive suboptimal pain management due to provider concerns about contributing to addiction or “medication-seeking” behavior (van Boekel et al., 2013). Providers’ narratives also revealed moralistic attitudes toward pregnant individuals with SUD, echoing findings from studies that associate pregnancy with heightened scrutiny and judgment (Terplan et al., 2015b).

Importantly, providers recognized the value of a whole-person approach to patient care, which acknowledges the broader context of a patient’s life, including past trauma, social stressors, and structural barriers to care. This perspective aligns with clinical empathy, a person-centered practice that involves actively listening, demonstrating understanding, and responding to patients with genuine compassion and care (Schwan, 2018). Clinical empathy goes beyond emotional resonance to appreciate a patient’s lived experience and how this shapes their emotions and behaviors in clinical interactions (Substance Abuse and Mental Health Services Administration, 2014). Providers’ recognition of the importance of listening, validating patients’ experiences, and avoiding judgment reflects core principles of trauma-informed care (Shank et al., 2024). By addressing bias and stigma through conscious reflection and unconditional positive regard, healthcare providers can build an authentic connection with patients to counteract the mistrust stemming from patient’s prior negative healthcare experiences and fears of punitive actions, such as child custody loss (Mahoney et al., 2019).

The role of communication in managing patients’ expectations, particularly regarding pain management and interactions with child welfare systems, was another notable finding. This reinforces previous studies that identify shared decision-making as a key factor in improving outcomes for patients with SUD (Bright et al., 2022). Providers recognized that transparent communication both alleviates patient fears and empowers them to engage in care. However, providers were sometimes unsure how to engage in that communication due to gaps in knowledge of both clinical management and SUD-related regulations. Increasing providers’ self-efficacy through education, training and support tools may in turn improve the quality of their communication with patients.

While many providers in our study expressed a desire to enhance their communication and ability to deliver person-centered, empathetic care to pregnant individuals with SUD, some conveyed ambivalence, frustration, or burnout, particularly when navigating unclear institutional protocols. Provider burnout in the face of resource scarcity was particularly salient in the context of rural healthcare. Providers described feeling overwhelmed by the dual challenge of limited resources and the complex needs of patients with SUD. These challenges align with existing research documenting the strain on rural healthcare systems, including geographic isolation, workforce shortages, and inadequate funding (Heitkamp and Fox, 2022). Participants emphasized the emotional toll of maintaining empathy while managing high patient acuity and systemic barriers that offer them limited support. Burnout was compounded by the lack of specialized services, such as addiction treatment programs and mental health resources, which are critical for comprehensive, evidence-based care. Additionally, mandatory reporting laws and inconsistent substance use screening protocols often place providers in ethically fraught positions, highlighting how punitive policies can overshadow supportive interventions.

Overall, providers acknowledged that structural pressures intensified their negative experiences and contributed to implicit and explicit biases in care delivery. To alleviate their frustrations and burnout at an individual level, some providers relied on peer support and mindfulness practices as resilience strategies, while others noted that systemic interventions such as structured debrief sessions and comprehensive training could help sustain provider well-being and enhance quality of care. By examining provider experiences within context of the healthcare system, our findings underscore the importance of aligning clinical training, institutional policies, and public health strategies to reduce stigma and promote equity in maternal care.

Providers’ stated gaps in knowledge highlight the need for ongoing clinical training. Uncertainty regarding best practices for managing pain and NAS, along with knowledge gaps in regulatory frameworks for caring for patients with SUD, emerged as significant barriers to effective care. Providers’ concerns about balancing adequate pain relief with the risk of misuse highlight a critical gap in SUD-specific training. Similar findings have been reported in studies emphasizing the need for standardized guidelines and continuing education on NAS and pain management in patients with SUD (Mahoney et al., 2019). Participants desired specific training to address these gaps and empower providers to manage the complexities of SUD in pregnancy with greater efficacy. Continuing education should prioritize non-opioid and opioid pain management, trauma-informed care, and care of infants with NAS to improve clinical confidence and reduce bias, ultimately improving outcomes for mother-newborn dyads affected by SUD. Specifically, simulation-based education and interdisciplinary team debriefings can support providers in practicing respectful communication, pain management strategies, and decision-making under stress.

Moreover, institutional policies that promote standardized child protective services reporting protocols, integrated plans of safe care, and clearer guidance on substance use screening versus testing are critical to ensure consistency, mitigate provider moral distress, and reduce disparities in care. These approaches can collectively strengthen provider preparedness and foster more equitable, evidence-based care for birthing individuals with SUD.

A major strength of this study is its focus on rural healthcare providers, a population often underrepresented in research on SUD in pregnancy. By capturing diverse perspectives across multiple clinical sites and specialties, the study provides nuanced insights into the unique challenges of rural care delivery. Additionally, the use of both FGDs and IDIs allowed for a comprehensive exploration of group dynamics and individual experiences. Despite the demographic skew in our sample, we observed thematic saturation across the major provider groups (nurses and physicians) included in the study, with recurrent patterns emerging within and across both rural and urban sites.

However, the study also has limitations. Findings are specific to Utah and may not generalize to other rural settings with different sociocultural or healthcare dynamics. Moreover, our sample was predominantly composed of nurses who identified as White, potentially underrepresenting perspectives from racially and ethnically diverse providers whose experiences with marginalized patients, navigation of systemic barriers, and interpretations of implicit and explicit bias may vary. Future research should aim to recruit a more racially diverse and role-balanced sample, including community-based social workers, doulas, midwives, and peer recovery coaches, to further explore variation in provider perspectives and experiences. Finally, the reliance on self-reported data and self-selection to engage with the research team introduces the potential for social desirability bias, which may be heightened in group discussions.

In conclusion, this study underscores the multifaceted challenges faced by healthcare providers in caring for pregnant individuals with SUD, and how these challenges are often exacerbated in rural settings. By illuminating the impact of stigma, resource limitations, and knowledge gaps, the findings highlight the need for targeted provider training, policy initiatives to better support people with SUD, investment in community resources, and institutional support mechanisms that promote non-stigmatizing, compassionate care for individuals with SUD during pregnancy.

## Supplementary Material

MMC1

MMC2

MMC3

MMC4

MMC5

## Figures and Tables

**Table 1:** Demographics of FGD and IDI Participants.

	FGD Participants	IDI Participants
		
Characteristic	N (%)	N (%)

**Total Participants**	**37 (100)**	**15 (100)**
**Role**		
Nurses	29 (78.4)	3 (20.0)
Midwives	1 (2.7)	1 (6.7)
Physicians	6 (16.2)	8 (53.3)
Social Workers	1 (2.7)	2 (13.3)
Peer Recovery Specialists	0 (0.0)	1 (6.7)
**Gender**		
Female	32 (86.5)	9 (60.0)
Male	5 (13.5)	6 (40.0)
**Race and Ethnicity**		
Asian/Pacific Islander	0 (0.0)	1 (6.7)
Black/African American	1 (2.7)	0 (0.0)
White/Caucasian	35 (94.6)	14 (93.3)
Prefer not to say	1 (2.7)	0 (0.0)
**Years of Experience**		
< 5 Years	9 (24.3)	4 (26.7)
5–10 Years	11 (29.7)	3 (20.0)
> 10 Years	17 (46.0)	8 (53.3)
**Location of clinical practice**		
Urban	4 (10.8)	9 (60.0)
Rural	33 (89.2)	6 (40.0)

## Appendix A. Supporting information

Supplementary data associated with this article can be found in the online version at doi:10.1016/j.drugalcdep.2025.112942.
