# Supplementary material for: Experiences of healthcare providers caring for pregnant individuals with substance use disorder
Source: Drug Alcohol Depend. Author manuscript; Available in PMC 2025 Dec 19. (PMC12716092; doi:10.1016/j.drugalcdep.2025.112942)
Supplement: MMC1 [file NIHMS2121200-supplement-MMC1.docx]

**INSPIRE Research Team**

| **Initials** | **Credentials** | **Occupation** | **Gender** | **Experience and Training** |
| --- | --- | --- | --- | --- |
| JB | MPH | Senior Qualitative Researcher | Female | 15+ years of experience in public health and mixed methods research |
| OH | MD (in progress) | Medical student | Female | Trained in public health and mixed methods research |
| AN | MSc | Researcher Coordinator | Female | 10+ years of experience in public health and mixed methods research. |
| KLB | MA | Research Fellow | Female | Trained in narrative medicine and qualitative research; mentored throughout the coding process. |
| SC | CNM, PhD | Faculty Clinician / MPI | Female | 20+ years of experience in simulation design, perinatal care, and mixed methods research. |
| MW | PhD | Faculty / MPI | Female | 20+ years of experience in public health and mixed methods research. |
