# Supplementary material for: Experiences of healthcare providers caring for pregnant individuals with substance use disorder
Source: Drug Alcohol Depend. Author manuscript; Available in PMC 2025 Dec 19. (PMC12716092; doi:10.1016/j.drugalcdep.2025.112942)
Supplement: MMC2 [file NIHMS2121200-supplement-MMC2.docx]

PROJECT INPSIRE:

KEY INFORMANT INTERVIEWS WITH HEALTH CARE PROVIDERS

The purpose of this interview is to learn about your experiences caring for individuals with substance use disorders who are giving birth. We will use this information to develop a training curriculum to support providers like you in caring for this patient population.

As covered in the consent form, our conversation today will be used for research purposes only. When we present the results, we will aggregate everything we hear so that no individual is identified. We hope you will feel comfortable giving your honest opinions and feedback. If any of my questions are unclear, please let me know and I can repeat or reword the question.

For this study, we define substance use disorder as compulsive use of drugs and/or alcohol that is distressing and leads to consequences including health problems, disability, and failure to meet major responsibilities at work, school, or home.

As we discuss your experiences caring for intrapartum patients with substance use disorders, I want to acknowledge that many people have personal experiences with SUD. Because personal and professional experiences are often intertwined, I invite you to reflect on both. Personal experiences can be difficult to talk about. Please feel welcome to share (or not share) personal experiences during the interview.

Do you have any questions before we begin?

Let’s get started!

1. ***Can you please describe your role(s) in the hospital?***
   1. *How are you involved in L&D?*
   2. *How frequently do you care for individuals with SUD during birth + postpartum?*
2. ***Tell me a story***

Can you tell me the story of when you provided care for a person with substance use disorder during labor, birth, or immediately postpartum. Take me back to that clinical encounter (who was the patient? Who was with her? Who was with you? What happened? How did you feel in the moment? After?)

1. ***Experience providing SUD intrapartum care***
   1. So, how does this process typically work—who is requesting the consult?
   2. When you arrive, how much of the conversation is about the patient’s SUD history and goals with pain management?
      1. Do you ever get to have conversations pre-operatively? If so, what conversations do you have about pain?
      2. Do you have patients concerned about opiates in the epidural
      3. What is the overlap between your work and the acute pain clinic?
   3. What are your conversations around expected pain level?
   4. Postpartum epidural? Can you tell me about when those are used?
   5. What specific protocols are in place (opioid sparing protocol, what about for patients with other types of SUD? What are the challenges you see post c-section for pain control in those patients?)
   6. Aside from the clear clinical differences in pain management, what is different about caring for patients with SUD vs. those who do not have SUD?
   7. What has surprised you about caring for patients with SUD?
2. ***Personal Experience, bias and stigma***
   1. How do you see bias and stigma show up in your facility? On your acute pain team?
      1. How does bias show up for you, personally?
   2. Do you think health care teams are less sympathetic to high reported pain from SUD patients?
3. ***How care could be improved***

Now that we have discussed how things *are*, I’d like to imagine how they *might be.*

- 1. If you could wave a magic wand and change one thing about intrapartum SUD care in your hospital, what would you change?
  2. What do you wish you better understood about caring for patients with SUD?
  3. What do you wish your colleagues better understood about caring for patients with SUD?

1. ***Training plans***
   1. After speaking with provider teams across the state, we are leaning towards developing a training specifically around how manage pain for mother during labor and postpartum.
      1. What clinical components do you think are most important and doable in low resource settings?
         1. For example, Do you think ketamine is a reasonable option for smaller hospitals around the state?
      2. What recommendations do you have for how to address provider bias as it arises in pain management?
      3. If you could give the lone anesthesiologist at a rural hospital one piece of advice about how to provide high quality care to pregnant/postpartum patients with SUD, what would you say?
2. Any other suggestions for our team as we design our training?

***This is the end of my questions.***

***Do you have anything to add on related to the care of birthing individuals with substance use disorders?***
