# Supplementary material for: Experiences of healthcare providers caring for pregnant individuals with substance use disorder
Source: Drug Alcohol Depend. Author manuscript; Available in PMC 2025 Dec 19. (PMC12716092; doi:10.1016/j.drugalcdep.2025.112942)
Supplement: MMC3 [file NIHMS2121200-supplement-MMC3.docx]

**PROJECT INPSIRE:**

**FOCUS GROUP DISCUSSION WITH HEALTH CARE PROVIDERS**

The purpose of today’s discussion is to learn about your experiences caring for patients with substance use disorders during the intrapartum period. The knowledge we gather today will be incorporated into a training curriculum to support future healthcare teams.

Our conversation will be used for research purposes only. Any findings we share will be anonymized and aggregated with other responses so that no individual is identified. We hope you will feel comfortable giving your honest opinions and feedback.

We define substance use disorder as compulsive use of drugs and/or alcohol that is distressing and leads to consequences including health problems, disability, and failure to meet major responsibilities at work, school, or home.

As we ask you to share about your experiences taking care of patients with substance use disorders who are in labor, giving birth, or during their hospital stay immediately postpartum, we want to acknowledge that many of us may also have personal experiences with SUD. Because personal and professional experiences are often intertwined, we invite you to reflect on both. Personal experiences can be difficult to talk about. You are welcome to share personal experiences in the group if you feel comfortable, or we can talk about these experiences later in an individual interview.

**Some ground rules for today’s discussion:**

- If any of our questions are unclear, please let us know and we can repeat or reword the question.
- Everyone’s experiences are different, and equally valid. Because the goal of this discussion is to understand a wide breadth of experiences, we would love to hear from everyone.
- If you feel uncomfortable answering some questions in front of the group and would like to share your experience in an individual interview later, please let us know after the discussion.
- Although we cannot guarantee anonymity in a group setting, we ask that everyone participating in the discussion today respects the confidentiality of what is shared here.

**Do you have any questions before we begin?**

**Introductions: Can you please share:**

- **Name**
- **Current position**
- **What interest you have in this research topic/why you decided to come today?**

**I am going to go ahead and start the recording. For the sake of the recording, the first time you answer a question, can you please share your role at the hospital (I will remind you to do so!)?**

1. ***Hypothetical Scenario***

Let’s start the discussion with a hypothetical patient scenario. The goal of this scenario is to understand your hospital processes and imagine what care of this patient might look like. We know in many hospitals staff members perform multiple roles or work in multiple departments. Please feel free to answer questions relevant to any of the hospital roles or areas where you work.

Here is the initial information about the patient’s presentation to the Emergency Department {Distribute Case Study Handout 1 to ED providers}

Can someone please read the case study out loud to the group?

Handout 1

Anna visits the emergency room because she has severe abdominal pain and leaking of fluid. From her history in ED you learn she is a G4P2012 who is 36 weeks pregnant. She discloses that during her pregnancy she has been in an outpatient treatment facility to manage her methamphetamine and opioid use disorder. Today her vital signs are stable, the FHR is 140, uterine contractions are moderate to palpation and every 10-12 minutes. She is nervous and asks “is my baby going to get taken away?”

**For the ED staff:**

- What thoughts might be going through your mind during your initial intake?
- How would you talk with Ana about her situation?
- How would you answer her question?
- What are your experiences caring for patients presenting to the ED in similar situations?

Next, let’s say Anna has been admitted to the Labor and Delivery Unit {Distribute Case Study Handout 2 to L&D providers}. Can someone please read the case study out loud to the group?

Handout 2

The ED staff has diagnosed PPROM, here is the transfer report you get from the ED staff. Anna is a 23 y.o. G3P2012 at 36w1d by 11 week u/s, with PPROM and early labor, with a cervical exam of 4/50/-3/clear fluid, GBS unknown. She has a h/o OUD and methamphetamine and has been on suboxone for 1 week. Anna states that the last time she used was 2 weeks ago, she is currently living in a transitional facility. Her pregnancy is otherwise non-complicated, her HIV/Hep B/HepC/RPR/CG/CT are negative. Her VS and labs today are normal, no evidence of infection, normotensive, FHR Cat 1.

Now let’s hear from the **labor and delivery providers:**

- What thoughts might be going through your mind in this situation?
- How would you talk with Anna about her situation?
- How would you answer if she asked “is my baby going to be taken away?”
- What are your experiences caring for patients during labor and birth who are in similar situations?

(Facilitator Reads this section out loud) Anna has had a vaginal birth of a viable infant with APGAR 8/8, the baby needed C-PAP for 5 mins, starting at 10 mins of life. The baby is stable now and transitioning in the nursery. You, the pediatric team has been asked to consult about Anna’s baby.

Now let’s hear from the **pediatric providers:**

- What thoughts might be going through your mind in this situation?
- How would you talk with Anna about her situation?
- How would you answer if she asked “is my baby going to be taken away?”
- What are your experiences caring for patients in similar situations?

**For Everyone:**

- What specific extra education would you do with Anna in the early postpartum period?

(Facilitator note: try to get at breastfeeding, DCSF reporting, etc.)

- What other professional team would you include in your discussions about or with Anna (e.g. social work, case workers, etc.)
- How is your care for patients with SUD different than for those without?
- What would you change about your current care of patients with SUD?

1. ***Frequency of care of birthing individual with SUD***

Thank you discussing this case. Now I’d like to switch gears from the hypothetical and talk about your actual clinical experience in caring for patients with SUD during labor, birth, or immediately postpartum.

- How frequently do you encounter a patient with a SUD in your inpatient clinical setting?
- What types of SUD are you seeing among patients who present in labor? (opioids, methamphetamine, alcohol, polysubstance use etc.)
- How do you know that a patient you are caring for has a SUD? (screening, confirmation, information in medical record, provider-to-provider report)
- Does your facility have guideline or protocols for care? What are your thoughts on those?

1. ***Attitudes related to caring for a birthing individual with SUD***

- How do you see bias show up in the care pregnant people with SUD receive?
- What do you wish you better understood about caring for patients with SUD?
- When you have a patient with SUD, what are your expectations about how the labor, birth, postpartum and breastfeeding will go?

So far we have discussed your experiences caring for patients with SUD. We know that many of us also have our own experiences with SUD in our families and communities. I'd like to give you space to share that here, to your level of comfort. (Give space, see what comes up for participants)

- How does your personal experience impact your work?

1. ***Burnout and resilience***

We know that health care providers have very difficult jobs. There is data to suggest that in recent years experiences of burnout have increased among healthcare workers. Burnout is defined as a state of emotional, physical and mental exhaustion caused by excessive and prolonged stress.

How common do you think burnout is among providers in your unit?

- What contributes to burnout in your unit?
- Does providing care for patients with more complex needs, such as patients with SUD, contribute to burnout in your unit?

The consequences of burnout include feeling emotionally disconnected from our patients, or more quickly becoming frustrated or angry with our patients.

- Do you think that provider burnout may contribute to neglect or sub-optimal treatment of patients with complex needs?
  - *If no*: Why not?
  - *If yes*: Do you think that provider burnout impacts the treatment of patients with substance use disorders?
    - Why do you say this?
    - What would this look like?
- What are things that help to prevent burnout? This could include things done at the institutional level, or things you do personally to prevent burnout.

1. ***Training***

- What formal training, if any, have you received on caring for an individual with an SUD during birth?
  - Medication for OUD
  - Pain management for patients with SUD
  - How to interpret a urine tox
  - Laws about SUD and pregnancy
  - Connecting patients to resources
  - If/when to contact DCFS
  - Bias training
  - Signs of methamphetamine toxicity (“over-amping”)
  - Recognizing impact of SUD in neonates (and how to differentiate from other health conditions)
  - Signs and treatment of neonatal abstinence syndrome/ Fetal alcohol syndrome
- In addition to formal training on the topic, where else are you learning about how to care for patients with SUD during labor, birth and immediate postpartum?
- What additional training would you like to receive on this topic?
- What suggestions do you have for our team as we design our training?
