# Supplementary material for: Experiences of healthcare providers caring for pregnant individuals with substance use disorder
Source: Drug Alcohol Depend. Author manuscript; Available in PMC 2025 Dec 19. (PMC12716092; doi:10.1016/j.drugalcdep.2025.112942)
Supplement: MMC4 [file NIHMS2121200-supplement-MMC4.docx]

Final coding template for provider experiences delivering maternal care to individuals with SUD

| Theme | Sub-Themes | Mapped Manuscript Theme |
| --- | --- | --- |
| Resiliency strategies | 1. Pride and ownership in their work 2. Teamwork 3. Structural strategies and resources 4. Recognizing and managing your own triggers | 1. Provider burnout amid resource scarcity and systemic barriers |
| Health Systems Structure | 1. Lack of coordinated care within and between systems 2. Understanding of hospital policies 3. Provider wearing lots of hats 4. Developments/advancements in the field | 1. Provider burnout amid resource scarcity and systemic barriers 2. Knowledge gaps in clinical management and SUD-related regulations. |
| DCFS | 1. Perception of hospital staff/relationship with DCFS 2. Perception by patients (as shared by staff) | 1. Perceptions of pregnant individuals with SUD 2. Building trust through supportive communication and empathy |
| Lack of resources | 1. No substance use programs locally 2. Resources not tailored toward individuals with complex situations (e.g., small children, gabapentin use, owning dogs) 3. Lack of resources causes frustration/burnout 4. What resources or policies currently exist | 1. Provider burnout amid resource scarcity and systemic barriers 2. Knowledge gaps in clinical management and SUD-related regulations. |
| Bias | 1. Prioritize baby’s health 2. Ideas for addressing bias | 1. Perceptions of pregnant individuals with SUD |
| Rural is different | 1. Small town vibe vs. big city resources 2. Don’t bring big-city solutions to rural areas 3. Rural stoicism / lack of trust within healthcare system | 1. Provider burnout amid resource scarcity and systemic barriers |
| Empathy | 1. Sees the patient’s whole story 2. Examples of unbiased language 3. Personal experiences | 1. Perceptions of pregnant individuals with SUD 2. Building trust through supportive communication and empathy |
| Clinical care | 1. Understanding of state laws regarding care 2. Protocols for how to care for patients with SUD 3. Pain and pain management 4. Desire for more / better pain management 5. Protocol for pain management 6. Desired training | 1. Perceptions of pregnant individuals with SUD 2. Knowledge gaps in clinical management and SUD-related regulations. |
